# Monitoring the Levels of Cellular NF-κB Activation States

**DOI:** 10.3390/cancers13215351

**Published:** 2021-10-26

**Authors:** Johanna Meier-Soelch, Christin Mayr-Buro, Jana Juli, Lisa Leib, Uwe Linne, Jan Dreute, Argyris Papantonis, M. Lienhard Schmitz, Michael Kracht

**Affiliations:** 1 Rudolf Buchheim Institute of Pharmacology, Justus Liebig University, 35392 Giessen, Germany; Johanna.Meier-Soelch@pharma.med.uni-giessen.de (J.M.-S.); Christin.Mayr-Buro@pharma.med.uni-giessen.de (C.M.-B.); Jana.Juli@pharma.med.uni-giessen.de (J.J.); Lisa.Leib@pharma.med.uni-giessen.de (L.L.); 2 Mass Spectrometry Facility of the Department of Chemistry, Philipps University, 35032 Marburg, Germany; linneu@staff.uni-marburg.de; 3 Institute of Biochemistry, Justus Liebig University, 35392 Giessen, Germany; Jan.Dreute@biochemie.med.uni-giessen.de; 4 Institute of Pathology, University Medical Center Göttingen, 37075 Göttingen, Germany; argyris.papantonis@med.uni-goettingen.de

**Keywords:** NF-κB, signaling, kinase, transcription factor, methods, single cell assays, EMSA, ChIP-seq, PLA, RNA-FISH

## Abstract

**Simple Summary:**

In stress and disease situations, cells must rapidly and in a coordinated manner change their gene expression patterns to respond adequately. The NF-κB system comprises five transcription factors that are released from the cytosol to enter the nucleus in response to a wide range of extracellular stimuli via a complex cytosolic signaling system. In the nucleus, activated NF-κB dimers bind to specific chromatin loci across the entire genome and induce the expression of a broad repertoire of genes that regulate immune and inflammatory responses. Consistent with its biological importance, the extent of NF-κB activity is regulated and controlled at multiple levels. The aim of this review is to comprehensively present and discuss the currently available conceptual and methodological approaches to monitor the molecular activation status of the NF-κB system, including multi-level single cell analysis.

**Abstract:**

The NF-κB signaling system plays an important regulatory role in the control of many biological processes. The activities of NF-κB signaling networks and the expression of their target genes are frequently elevated in pathophysiological situations including inflammation, infection, and cancer. In these conditions, the outcome of NF-κB activity can vary according to (i) differential activation states, (ii) the pattern of genomic recruitment of the NF-κB subunits, and (iii) cellular heterogeneity. Additionally, the cytosolic NF-κB activation steps leading to the liberation of DNA-binding dimers need to be distinguished from the less understood nuclear pathways that are ultimately responsible for NF-κB target gene specificity. This raises the need to more precisely determine the NF-κB activation status not only for the purpose of basic research, but also in (future) clinical applications. Here we review a compendium of different methods that have been developed to assess the NF-κB activation status in vitro and in vivo. We also discuss recent advances that allow the assessment of several NF-κB features simultaneously at the single cell level.

## 1. Introduction

The NF-κB pathway has been discovered 36 years ago and its broad role in diverse (patho)physiological responses has sparked a tremendous and ongoing interest to determine its activation status in cells from healthy or diseased individuals [1,2]. The NF-κB signaling pathway regulates the localization and activity of NF-κB transcription factors, which are found in all nucleated cells. In general, the NF-κB system plays an important role in multiple processes that require the induction of appropriate gene responses in order to cope with all types of stressful conditions, but some tissues or cell types also use this pathway for constitutive functions [3].

In this review, based on many years of experimental work in the Schmitz and Kracht laboratories, we attempt to give a comprehensive overview on methods employed to study the NF-κB pathway and discuss some of their intrinsic and conceptual limitations. We refer to three layers of NF-κB activation that need to be considered in order to correctly interpret the cellular NF-κB activity status and its functional implications. We use published and some unpublished preliminary data to illustrate the outcome of typical experiments and briefly discuss the advantages or disadvantages of individual assays used in the field to monitor NF-κB activity.

### 1.1. Brief Description of the Core NF-κB Pathways and Their Components

The broad array of NF-κB-activating stimuli includes more general adverse signals such as DNA damage, lipid peroxidation, free DNA or RNA, and UV radiation, to name only a few. In addition, the stimuli also comprise specific signals that operate through plasma membrane receptors such as proinflammatory cytokines (IL-1α or IL-1β, TNFα), lipopolysaccharide (LPS), and T-cell co-stimulation [4]. Downstream of receptors, the various cytosolic activation pathways have in common that they involve the inducible formation of protein/protein interactions, which are controlled by highly regulated post-translational modifications, primarily phosphorylation and ubiquitination [5,6].

Ultimately, all the different cytosolic NF-κB activating pathways converge in the generation of active, DNA-binding competent dimers, which translocate to the nucleus and function to reprogram gene expression. The NF-κB family of transcription factors is composed of five different DNA-binding proteins, namely p65 (*RELA*), p50 (*NFKB1*), p52 (*NFKB2*), c-REL, and RELB which form up to 15 different homo- or heterodimers [7]. The family members NF-κB1 (also known as p105/p50) and NF-κB2 (also known as p100/p52) are derived from precursor proteins, either during translation or through phosphorylation-induced partial proteolysis to yield the DNA-binding forms p50 and p52, respectively [8].

The immunologically relevant induction steps of NF-κB proceed via the canonical and noncanonical NF-κB signaling pathways, while DNA damage-triggered NF-κB activation employs the atypical activation pathway [2]. The canonical and atypical pathways lead to the activation of a complex signaling cascade including the ubiquitin E3 ligase TRAF6 and the IκB kinase (IKK) complex which phosphorylates the inhibitor of NF-κB (IκB) proteins [9,10]. The phosphorylation enables subsequent ubiquitination and proteasomal degradation of the IκB proteins, namely IκBα, IκBß, and IκBε [11,12]. This in turn leads to the release, nuclear translocation, and DNA-binding of NF-κB in order to induce gene expression. In the non-canonical activation pathway, the free NF-κB dimers are generated by IKKα-mediated phosphorylation of the p100/NF-κB2 precursor protein, which leads to its processing to p52 and the generation of p52/RelB and p50/RelB dimers [13,14].

Acute NF-κB activation is typically terminated by induction of a complex network of negative feedback loops that occur with a characteristic time delay, thereby permitting full NF-κB function during the interim period [15]. The most prominent examples are the NF-κB driven re-synthesis of IκBs and the induction of TNFAIP3/A20, a negative regulator of the IKK complex [16,17,18,19]. These autoregulatory negative feedback loops reset the pathway to its latent state, but may function inappropriately in situations of chronically elevated “low-ON” NF-κB activity, as it occurs in pathophysiological situations including chronic inflammation or cancer (see below).

### 1.2. The Concept of Three Layers of NF-κB Activity

**Figure 1:**
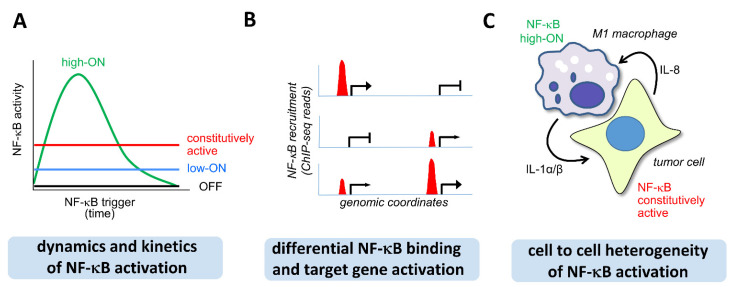
The three layers of NF-κB activation states. (**A**) Schematic representation of different modes of NF-κB activity. (**B**) Schematic showing various possibilities of NF-κB binding to specific genomic loci and the functional consequences on transcription. (**C**) Cellular heterogeneity of NF-κB activation, as exemplified by the interaction between a tumor cell and a neighboring macrophage.

The first layer of NF-κB activity refers to the dynamic nature of the NF-κB system, which is never static and rather characterized by various activation states, as schematically shown in Figure 1A. (i) In the constitutively active state, significant NF-κB activity is found in specific cell types such as Sertoli cells, B cells, hair follicle cells, and neurons [20,21,22,23,24,25,26]. (ii) In the “high-ON” state, most of the NF-κB dimers are rapidly released from inhibitory IκB proteins and translocate to the nucleus. The “high-ON” state is typical for fast and transient activation of the NF-κB system in response to proinflammatory triggers. (iii) In the “low-ON” state NF-κB shows a continuous low grade activation, as it often occurs in situations of smoldering inflammation or in the tumor microenvironment [26,27,28,29,30,31]. (iv) In the “OFF-state”, NF-κB is quantitatively trapped by IκB proteins in the cytosol and NF-κB-driven transcription is largely absent.

The second and possibly most important layer of regulation refers to the gene specific recruitment of NF-κB (Figure 1B). While the cytosolic NF-κB activation steps lead to the (global) liberation of DNA-binding dimers, the activity of this transcription factor is also regulated by a less well-understood nuclear pathway. An important general question in this context is how NF-κB gene specificity is ultimately achieved. Early studies suggested that the human genome contains around 300,000 NF-κB binding sites composed of the typical DNA sequence motif 5′-GGGRNWYYCC-3′ (R, purine; W, A or T; Y, pyrimidine) [32,33]. The large number of ChIP-seq results deposited in the encyclopedia of DNA elements (ENCODE) data base show that in most biological situations, only a fraction of all sites available in the genome is occupied by NF-κB [34]. However, neither the signals and mechanisms that determine the relative amount of nuclear NF-κB in stable association with specific genomic regions nor the signaling pathways regulating nuclear NF-κB activity have been defined [35]. Apparently, NF-κB binding is enabled by altered accessibility of chromatin regions facilitating the access of this transcription factor [36,37,38]. In this model, high affinity NF-κB sites are occupied once other nuclear proteins such as pioneering factors, chromatin remodellers or histone-modifying enzymes have relaxed nucleosome density to expose the NF-κB motifs and allow access of NF-κB subunits [37,39,40,41].

The third layer of NF-κB activity involves a remarkable cell to cell variability (Figure 1C). Single cell assays have revealed that activation of NF-κB and the resulting gene expression patterns can be differentially phased and are highly variable [42,43]. It is possible to find cells with low levels of NF-κB activity adjacent to cells with high activity and strong activation of NF-κB target genes [44,45,46]. As many of the latter are secreted proteins, such a behavior may profoundly shape the overall tissue response. This phenomenon implies that conclusions derived from population-based assays can be potentially misleading and accordingly we will highlight below some approaches to reveal the NF-κB activation status at the single cell level.

**Figure 2:**
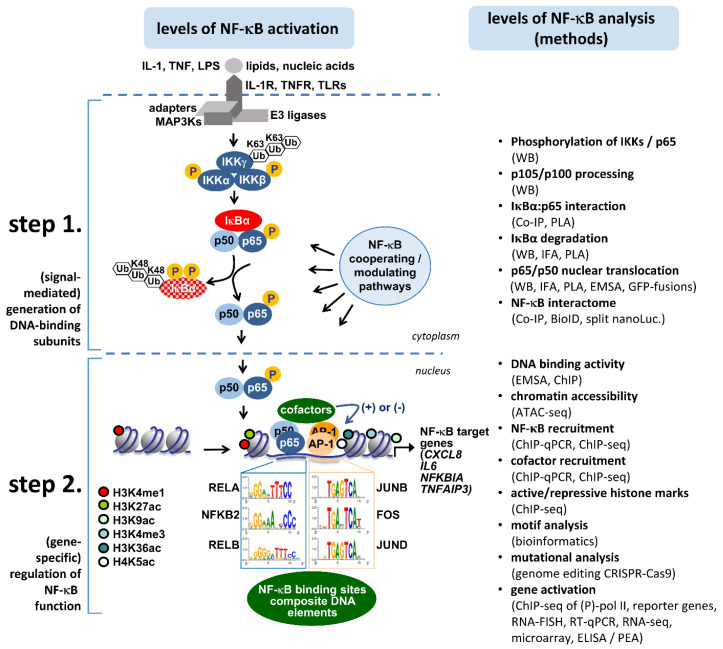
The two intracellular levels of NF-κB activation and the approaches to determine NF-κB activity. Overview of the core components and features of the canonical NF-κB pathway, its two regulation levels in the cytosol (step 1) and the nucleus (step 2), and the approaches and methods available to assess the NF-κB activation state. For details, see text.

As shown in Figure 2 at least a dozen different methods are available to assess the various intracellular regulatory levels of the NF-κB signaling pathways, which can be divided into a cytosolic (step 1) and a nuclear (step 2) part. Historically, primarily the cytosolic activation pathway has been investigated by a range of well-established methods to detect NF-κB activity. These primarily biochemical assays are still very important, as they yield information on the signal-mediated generation of DNA binding units. To reveal the gene-specific consequences beyond step 1, it is necessary to employ additional approaches. The advent of modern genomics and next generation sequencing (NGS) techniques has caused a shift toward methods that determine the direct occupancy of DNA by NF-κB and cooperating transcription factors at the genome-wide level [33,47,48,49,50,51,52,53]. These methods also deliver information about the status of the surrounding chromatin environment (cofactors, chromatin accessibility, histone modifications). Together with functional assays, they identify the regulatory modes directly controlling NF-κB target genes and thus yield context-specific information on NF-κB activation [54]. It is also important to bear in mind that NF-κB activation rarely occurs in isolation, as typically other stress pathways are activated in parallel. Three prominent examples are the p38, JNK, and JAK kinase pathways, which regulate the expression levels and activities of a large number of transcription factors, such as AP-1 and STAT proteins [55]. These pathways cooperate with the NF-κB system at multiple levels and can profoundly shape the amplitude and duration of the expression of NF-κB target genes [55].

## 2. Determination of NF-κB Activity

### 2.1. Detection of NF-κB Pathway Proteins

Determination of NF-κB activity requires careful consideration of two principal caveats that may complicate interpretation of the results: (I) The relative activation level of the pathway can be highly variable, as shown in Figure 1A. The NF-κB activity levels in the OFF state or the late “high-ON” state after termination of the NF-κB response are usually too low to be reliably detected. In contrast, the other activity stages can be accurately and usually quantitatively determined using a large arsenal of in vitro and in vivo methods. (II) The activation kinetics of the NF-κB system is highly dynamic and depends on many parameters. While activation in response to inflammatory stimuli and DNA damage occurs within minutes, induction of the non-canonical pathway and proteolysis of p105 and p100 takes several hours [56]. The same is true for the expression of NF-κB target genes, which can be induced with highly variable expression and induction kinetics [27,57,58,59]. In the following, we discuss in detail the different approaches and methods to monitor the *status quo* of NF-κB activation states, without considering methods that serve to explore the molecular mechanisms and regulatory functions exerted by this transcription factor itself.

**Figure 3:**
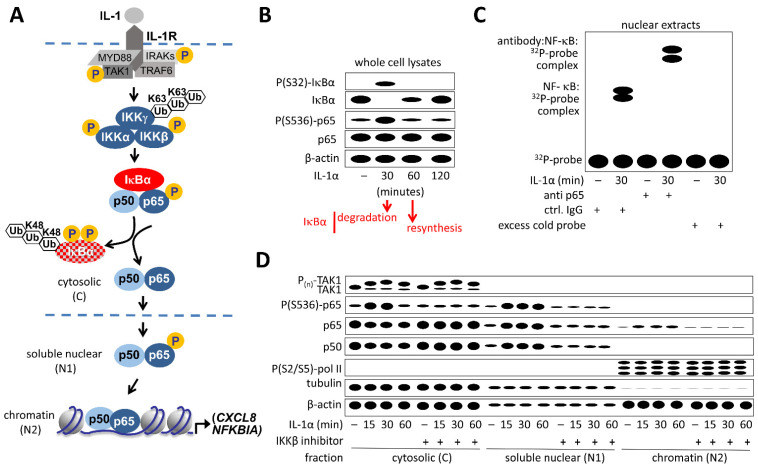
Key assays to determine NF-κB activation. (**A**) Overview of components and steps of NF-κB activation that can be monitored in cell extracts. (**B**) Typical pattern of protein bands observed by Western blot analysis of whole cell extracts of IL-1α-treated HeLa cells. A prototypical Western blot result of this kind is presented in Appendix Figure S5 of [62]. (**C**) Typical patterns of NF-κB protein complexes bound to labelled double-stranded oliogonucleotides containing NF-κB sites in vitro. Nuclear extracts from untreated and IL-1α-treated cells are mixed with probes and the resulting complexes are resolved by electromobility shift assays (EMSAs). The identity of proteins and the specificity of DNA binding is further determined by addition of NF-κB-specific antibodies (resulting in supershifts) and by competition of signals with unlabeled (“cold”) oligonucleotide probes. The EMSA is very sensitive to detect nuclear translocation of NF-κB upon cellular activation, but does not reveal the native genomic binding sites and targets as compared to ChIP. For typical EMSA results see [63,64]. (**D**) Subcellular distribution of NF-κB subunits determined by Western blotting of cytosolic, soluble nuclear and insoluble (chromatin-associated) fractions. The scheme shows the patterns of protein bands demonstrating that in IL-1α-activated KB cells (a HeLa subclone) around 30–50% of total p65 is rapidly translocated to the nucleus and a small part is stably associated with chromatin. In the same extracts, p105 processing is assessed by detecting p50, which also translocates to the nucleus. The addition of an IKKß-specific inhibitor (PHA-408) results in suppression of IL-1α-inducible (but not constitutive) p65 phosphorylation at Ser536 as well as inducible nuclear translocation of both, p65 and p50. In line with the model shown in (**A**), IKKß inhibition does not affect the activation of the upstream kinase TAK1, as determined by retarded electrophoretic mobility of the multi-site phosphorylated forms P_(n)_. For the original Western blots and quantification of the relative amounts of NF-κB subunits before and after activation, see Figure S4 of [65]. This type of experiment provides comprehensive and quantifiable information on multiple steps of NF-κB activation, but requires large numbers of cells and takes relatively long.

As shown in Figure 3A, most cell types express the core NF-κB pathway components, which can be readily determined by Western blotting (WB) or mass spectrometry-based proteomics (see below). As many inducers of the canonical and atypical NF-κB pathways (such as IL-1) lead to the proteolytic destruction of IκB proteins within 10 to 30 min of activation [11], the stimulus-induced decrease of these proteins is a key parameter to detect NF-κB activation. The time-resolved analysis of IκB expression also allows following its re-synthesis, which contributes to the termination of the NF-κB response [60,61] (Figure 3B).

Cells with constitutively active NF-κB such as B cells are characterized by elevated turnover of endogenous IκBα and IκBß, a feature that can be measured by determining their quantity at various time points after inhibition of de novo protein synthesis [66]. Activation of the non-canonical NF-κB pathway is assessed by changes in the ratio between p52 or p50 and their p100/p105 precursors, respectively. As an example, prolonged stimulation of cells with LPS was shown to trigger a conversion of p105 to p50 [67]). Active signaling generates increasing amounts of the DNA-binding proteins at the expense of the larger unprocessed forms [13,14]. The determination of protein amounts can also be very instructive for slowly occurring regulatory processes, as illustrated by the example of the progressively declining protein levels of IKKβ, NEMO/IKKγ, and IκBα in liver cells infected with human coronavirus 229E for periods ranging from 6 h to 24 h [68].

### 2.2. Detection of DNA-Bound NF-κB

As pointed out above, a characteristic hallmark of NF-κB activation is the generation of DNA-binding dimers, which translocate to the nucleus upon activation. Many assays measuring NF-κB activation are based on the finding that IκB-associated NF-κB subunits do not bind to their cognate DNA [69], implying that the DNA-binding activity of NF-κB is a good proxy for its activation status. DNA-binding can be measured by various in vitro methods including electrophoretic mobility shift assays (EMSAs), which separate free oligonucleotides containing a κB binding site from the slower migrating form in complex with bound NF-κB. These assays also allow to perform supershift assays with specific antibodies in order to determine the subunit composition of the shifted complex and can be combined with oligonucleotide competition assays in order to determine the impact of DNA sequence on binding affinities [70] (Figure 3C). EMSA experiments have been extended to EMSA-sequencing (EMSA-seq), where purified NF-κB proteins are incubated with degenerate oligonucleotide libraries, followed by EMSA and next generation sequencing of bound DNA molecules to determine binding site specificities [71]. The EMSA experiments can be performed with radiolabeled and non-radiolabeled oligonucleotides, but very often researchers now use alternative, ELISA-based approaches where extracts containing free NF-κB are incubated with oligonucleotides that are attached to a plate. Incubation of these oligonucleotide-coated plates with NF-κB containing extracts facilitate systematic and high-throughput binding studies of this transcription factor and antibody-based detection and quantification of DNA-bound NF-κB [72].

### 2.3. Detection of Post-Translational Modifications of NF-κB Pathway Components

The critical involvement of post-translational modifications can be used to monitor individual signaling steps with the help of modification-specific antibodies. These recognize different features such as phosphorylation of IκBα or IKKα/ß, thus allowing to monitor IKKα/ß activity independent from in vitro kinase assays. A variety of modification- specific antibodies are available from different commercial sources, but it is probably worth mentioning that a phospho-specific antibody determining p65 Ser536 phosphorylation is not a good proxy for NF-κB activity [73] and rather reflects the activation status of the responsible IKK kinase or other kinases targeting this site [74,75]. IκBα phosphorylation at Ser32 and Ser36 is often detected along with the drop in protein levels, whereas detection of K48-linked polyubiquitinated IκBα usually requires the blockade of the proteasome by MG132 or related compounds in order to stabilize the modified degradation products [76].

As shown in Figure 3B,D, experiments addressing the protein expression and post-translational modifications of NF-κB components are typically performed by Western blotting and thus require a relatively high number of cells. However, quantitative analysis of protein levels can also be done for smaller cell numbers by liquid chromatography coupled to mass spectrometry (LC-MS/MS), a method that has been tremendously improved and nowadays allows determining protein concentrations with unprecedented sensitivity. In addition, protein expression and post-translational modifications of NF-κB components can also be measured at the single cell level by quantitative immunofluorescence analysis.

Many proteins participating in NF-κB activation have intrinsically disordered regions, which are engaged in conformational switches [77]. A TNF-inducible conformation switch has also been shown for p65, as revealed by limited proteolysis assays and the use of a conformation-specific monoclonal antibody that preferentially immunoprecipitates the inducibly refolded p65 protein [78]. The use of conformation-specific antibodies has enabled great progress for studies on the p53 transcription factor [79] and it would be interesting to develop further antibodies for NF-κB research to image dynamic events in NF-κB signaling. This is important as mutations of the two intrinsically disordered C-terminal transactivation domains TA1 and TA2 of p65/RELA, which are subject to multiple phosphorylation events, profoundly shape the NF-κB-dependent transcriptome [80,81,82].

### 2.4. Detection of Subcellular Localization of NF-κB

**Figure 4:**
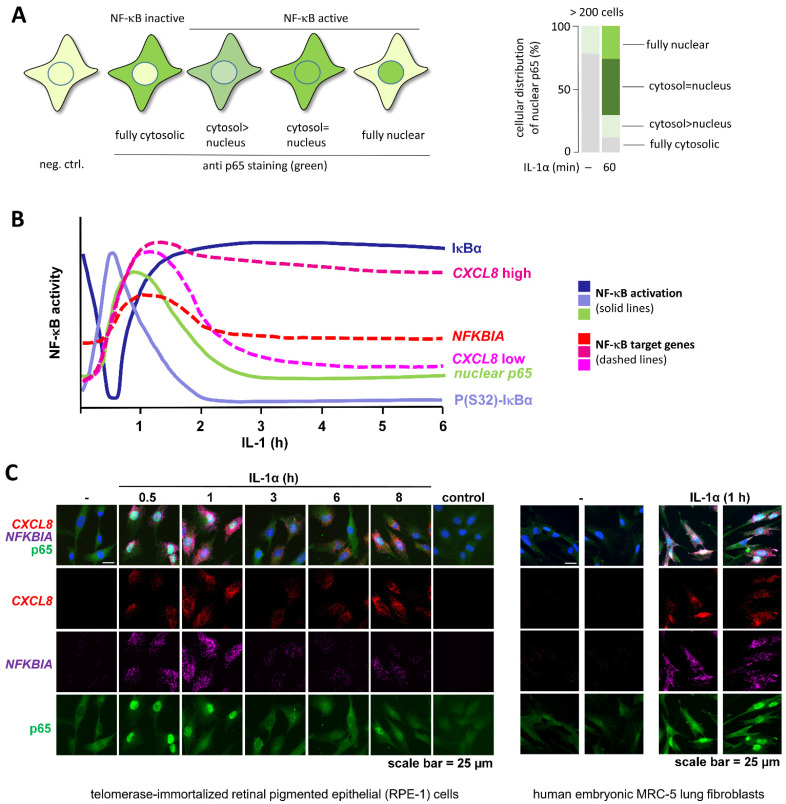
Key assays to determine heterogeneous NF-κB activation states in individual cells. (**A**) Immunofluorescence analysis (IFA) of p65 translocation. Similar to the Western blots shown in Figure 3D, this assay detects the overall nuclear translocation of p65, but also reveals substantial cell to cell heterogeneity which can be categorized and quantified. In this case, cells lacking a nuclear p65 signal were defined as “NF-κB pathway inactive”, whereas cells with nuclear signals show varying degrees of NF-κB pathway activation. The bar graph shows the heterogeneity of NF-κB nuclear translocation across at least 200 HeLa epithelial carcinoma cells. The original data were published in ([62], Appendix Figure S4). (**B**) Scheme showing the time-wise phasing of signaling to IκBα, the nuclear translocation of p65 and the subsequent induction of NF-κB target genes (at the examples of *NFKBIA* and *CXCL8* mRNAs). (**C**) IFA (for p65 protein, green) combined with smRNA-FISH for *NFKBIA* (purple) and *CXCL8* (red) mRNAs. The results demonstrate the single cell variability of basal and inducible nuclear translocation of p65 and the subsequent induction of the two mRNAs in human diploid epithelial cells or fibroblasts. smRNA-FISH was performed as described [62].

The inducible nuclear translocation of NF-κB subunits can be readily measured by a number of techniques including subcellular fractionation, where DNA-binding subunits are detected in the soluble nuclear fraction and to a minor extent also in the insoluble chromatin fraction (Figure 3D). Alternatively, and perhaps more common, nuclear translocation of NF-κB subunits can also be assessed by immunofluorescence [65,83,84]. Microscopy allows monitoring the single cell variability of the NF-κB activation state, as shown in Figure 4A. However, to fully appreciate the biological consequences of NF-κB activation, we recommend combining methods to detect changes at the protein level and the subsequent changes at the mRNA level within the same cell in order to track the true phasing and temporal activation kinetics of the entire NF-κB system (Figure 4B). Figure 4C shows two examples from diploid, nonmalignant, adherent human cell types in which nuclear translocation and target gene activation were measured simultaneously by Immunofluorecence (IF) and single molecule RNA Fluorescence In-Situ-Hybridization (smRNA FISH) experiments using a triple color approach. Immunofluorescence also allows to reveal NF-κB localization to subnuclear regions such as the nucleoli where the p65 subunit accumulates after induction of nucleolar stress [85] or to cytoplasmic inclusion bodies, as it occurs for example after infection of cells with respiratory syncytial virus [86]. Additionally, the fusion of NF-κB DNA-binding subunits to fluorescent proteins has been extensively used to study the dynamic oscillations of active NF-κB in living cells [43,44,46,87,88,89,90]. Further options include combined imaging and flow cytometry instruments (Amnis^®^ imaging and flow cytometry systems, Luminex, Austin, TX, USA) which facilitate the quantitative analysis of NF-κB nuclear translocation in heterogenous cell populations.

### 2.5. Detecting NF-κB Activation by Changes in Protein/Protein Interactions

**Figure 5:**
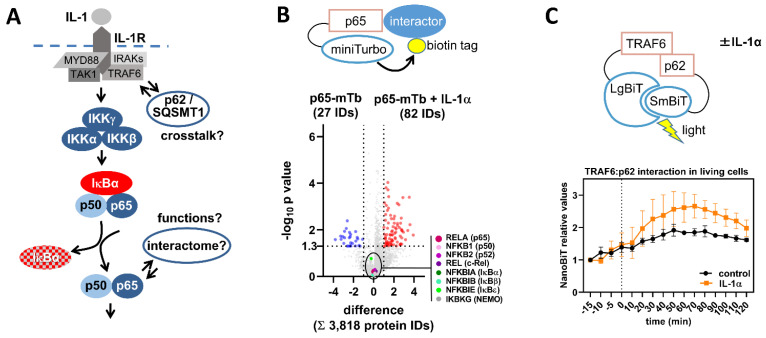
Global or pairwise monitoring of protein/protein interactions in the NF-κB system in living cells. (**A**) Scheme showing established and putative protein/protein interactions of the canonical NF-κB pathway. (**B**) The scheme shows the p65-miniTurbo (p65-mTb) fusion protein that was transiently transfected in HeLa cells using a doxycycline-inducible vector system. Expression was induced by 1 µg/mL doxycycline for 18 h. 24 h after transfection, cells were incubated in 50 µM biotin for 70 min and were left untreated or were stimulated with IL-1α for the last 60 min. Biotinylated proteins were purified from cell extracts using streptavidin-agarose beads and identified and quantified by LC-MS/MS using a TimsTOF system. The Volcano plot shows the differences of the mean protein intensities on the *x*-axis and the results of Student’s T-test on the *y*-axis comparing untreated with IL-1α-treated conditions. Mean protein intensities were determined from two biological and three technical replicates per condition. Out of 3818 proteins identified in total, 109 were found to differentially bind to p65-mTb upon IL-1α treatment (based on a two-fold cut-off and a *p* value of -log_10_ (*p*) ≤ 1.3, red and blue colors), while p65 and seven further components of the canonical NF-κB pathway were readily detected and evenly present in samples from unstimulated or stimulated cells. This approach holds great potential to comprehensively uncover stable, transient, and weak interactions of NF-κB components with cellular proteins in healthy or diseased conditions. (**C**) HEK293T-*IL1R1* cells, stably expressing the IL-1 receptor type 1, were co-transfected with doxycycline-inducible vectors encoding TRAF6-LgBiT and p62-SmBiT. Protein-expression was induced by doxycycline (1 µg/mL) for 30 h. Promega NanoBIT Live Cell Substrate^®^ was added to allow detection of luciferase activity in living cells. After 15 min of baseline measurement (minute = 0, dotted line), IL-1α (10 ng/mL) was added to three of the dishes as shown (yellow), while further three dishes remained untreated (black). The data were normalized to the first measurement and presented as means (*n* = 3).

As shown in Figure 5A, the major proteins in the NF-κB activation pathway engage in regulated protein/protein interactions, as exemplified by the loss of the IκB/NF-κB interaction after pathway activation or inducible recruitment of signaling proteins to cell surface receptors [91]. Moreover, upstream regulators such as TRAF6 can simultaneously regulate other key signal proteins and interact for example with p62/SQSTM1 to balance IL-1 signaling to NF-κB [92]. Additionally, IKK kinases and the NF-κB subunits itself constantly interact with other factors in the cell [93]. Traditionally, the network of regulated protein/protein interactions was determined by co-immunoprecipitation (Co-IP) experiments, but improved methods are now available to expand the sensitivity and the coverage of these analyses and, more importantly, to also capture weaker or transient interactions as they occur in living cells. These methods can be based on proximity labeling, where proteins are fused to an enzyme such as a Biotin ligase (e.g., BirA or miniTurbo) which in turn allows biotinylation of all interactors in the vicinity of the fusion protein (BioID, [94,95,96,97]). Streptavidin-coated beads are then used to enrich the entirety of the biotinylated interactors, which can be identified by Western blotting or mass spectrometry.

Proximity labeling approaches are less prone to in vitro artifacts, which can occur by disruption of cellular compartments upon cell lysis, thus potentially causing binding between factors, which are normally well separated and not interacting in the intact cell. Figure 5B provides an example of the proximity-labeling based analysis of the interactome of a p65-miniTurbo fusion protein. Using LC-MS/MS analysis, out of 3818 Streptavidin-purified proteins, we not only reliably found seven components of the core NF-κB pathway binding to the p65 fusion protein, but also identified more than 100 proteins that interact with p65 in an IL-1α-regulated manner. BioID experiments have high sensitivity for the detection of new NF-κB interactors, but always require validation of putative interactors by orthogonal approaches to identify false positives, which may result from background biotinylation.

In this regard, interesting pairs of protein/protein interactions can be dynamically and quantitatively studied employing a split NanoLuciferase system [98]. Fusion of potentially interacting proteins to two different subunits of the NanoLuciferase is used to allow complementation of both luciferase fragments, followed by the instant generation of light as a read-out for interaction as it occurs in the living cell. To test the functionality of this system for the analysis of dynamic NF-κB signaling, we fused the TRAF6 protein to a large 18 kDa portion of NanoLuciferase (termed large LgBIT) and its known interactor p62/SQSTM1 to a small 1.3 kDa fragment (termed small SmBIT). Tet-inducible expression of both proteins allowed detecting the constitutive interaction as well as the IL-1α-triggered increase of the TRAF6/p62 interaction and its subsequent dissociation in living cells (Figure 5C). As shown by these examples, it can be expected that, in future, such techniques will substantially broaden our current view on the (dynamic) NF-κB interactomes.

### 2.6. Detection of NF-κB-Binding in the Chromatin Environment

**Figure 6:**
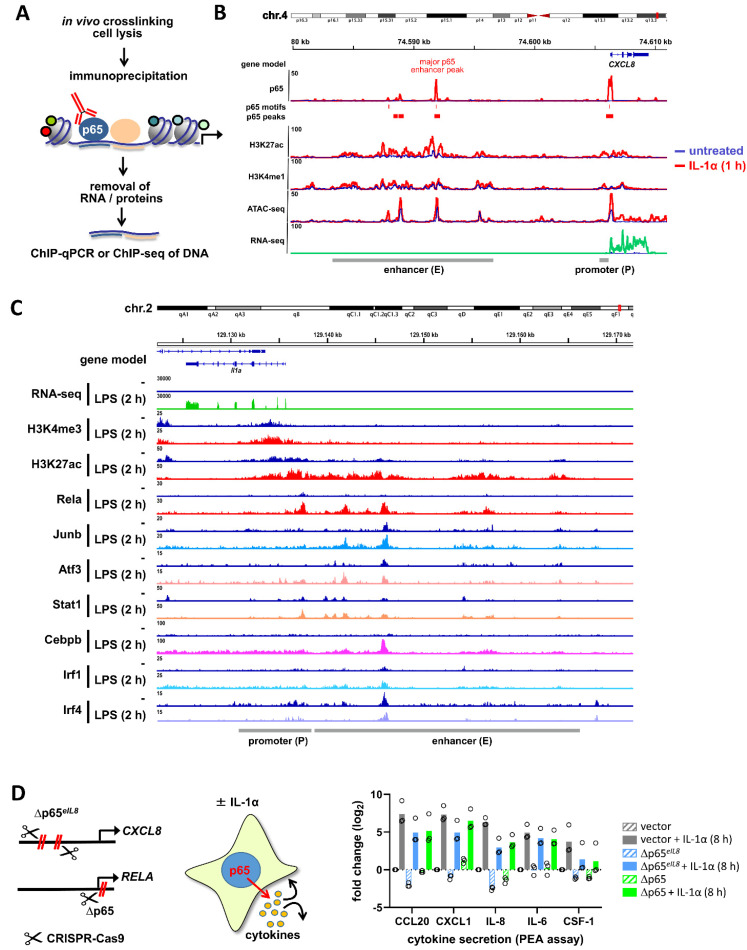
ChIP-seq, ATAC-seq, RNA-seq and genome editing to monitor the nuclear NF-κB pathways and their relevance for gene expression. (**A**) Principle of ChIP-qPCR or ChIP-seq assays. (**B**) Integrative genomics viewer (IGV) genome browser views of combined ChIP-seq, ATAC-seq, and RNA-seq analyses of the extended human *CXCL8* locus before (blue profiles) and after short-term stimulation with IL-1α (red, green profiles) to reveal (i) the genomic binding sites of p65 to enhancers and promoters, (ii) the presence of active enhancers (defined by inducible H3K27ac and constitutive H3K4me1), (iii) the regions of open or accessible chromatin (by ATAC-seq), and (iv) the resulting mRNA expression of the NF-κB target gene *CXCL8*. The figure was generated using published data from our laboratories [62,65]. The data can be retrieved from NCBI GEO (https://www.ncbi.nlm.nih.gov/geo, accessed on 12 October 2021) with the accession numbers GSE64224 and GSE52470. The function of the largest p65 peak in the enhancer was analyzed by genome editing as shown in (**D**) and described in detail in [62]. (**C**) Co-recruitment of multiple transcription factors together with NF-κB to the murine *Il1a* enhancers and promoters demonstrating the signal integration of the canonical NF-κB pathway with transcription factors activated by multiple other pathways at the level of chromatin. Samples were derived from LPS-stimulated murine dendritic cells, the figure was produced from ChIP-seq and RNA-seq data deposited in GSE36104 (published in [50]). H3K4me3 is indicative for actively transcribing genes and their promoters. (**D**) HeLa cells were genome-edited by CRISPR-Cas9 to generate < 60 nt microdeletions of the major NF-κB binding site in the human *IL8* enhancer (Δp65*^eIL8^*) or to deplete p65 protein expression due to a frame-shift in the first exon (Δp65). Supernatants of cells were analyzed before and after 8 h of IL-1α stimulation for the secretion of proteins encoded by five NF-κB target genes using PEA technology. Mean relative changes plus individual values from three independent experiments are shown. The cell lines are described in detail in [62]. Note the strong suppression of IL-8 (encoded by the *CXCL8* gene) and the variable quantitative requirement of NF-κB for basal or inducible protein secretion of further factors. (**B**,**C**) Numbers show read counts.

NF-κB also binds to its cognate DNA-binding site within the intact cell where the nuclear DNA is contained in chromatin and wrapped around the nucleosomes. Depending on the accessibility of binding sites, the NF-κB dimers can bind to thousands of sites in the genome. This is frequently investigated using chromatin immunoprecipitation in combination with qPCR (ChIP-qPCR) or ChIP coupled to sequencing (ChIP-seq) experiments. In a nutshell, this experimental approach relies on the chemical crosslinking of NF-κB to the DNA, followed by cell lysis and shearing of genomic DNA. Specific antibodies are then used to immunoprecipitate DNA-bound NF-κB, followed by purification and qPCR-mediated quantification of the co-precipitated DNA [99] (Figure 6A). If performed for a number of known NF-κB binding sites or even at the genome-wide level, this technique is the method of choice to reveal basal or stimulus-induced NF-κB DNA-binding in intact cells. Although this approach is very powerful, it is not without limitations. Despite the continuous development of protocols, ChIP-qPCR and ChIP-seq require considerable numbers of cells and the availability of high quality ChIP-grade antibodies, which can be a potential problem for some of the DNA-binding subunits. ChIP-qPCR experiments depend on a priori information on the NF-κB-bound genomic binding sites, which differ depending on the organism and cell type [54,100]. However, new techniques measuring chromatin association of transcription factors including CUT&RUN or CUT&TAG have been recently established [101,102]. As these techniques function with low cell numbers at high resolution they will soon be widely used also in the NF-κB field. Besides its ability to associate with DNA, IκBα-depleted NF-κB can also be detected with a monoclonal antibody recognizing an epitope covering the nuclear location signal (NLS) of p65. Structure analysis showed that this site is covered by the IκBα protein [103], thus allowing to use this antibody (designated alpha-p65MAb) to detect the free and uncomplexed p65 protein [104].

Figure 6B provides an example of ChIP-seq, ATAC-seq, and RNA-seq data derived from human epithelial carcinoma cells which were combined to reveal four regions of strong IL-1α-inducible p65 binding (three of which contain a predicted NF-κB motif) to the inducible enhancers and promoters of the prototypical NF-κB target gene *CXCL8* (*IL8)* [62,65]. Enhancers and promoters show already constitutively open and some inducible regions with increased chromatin accessibility based on ATAC-seq peaks, a transposase-based approach that yields information on chromatin accessibility [105,106].

Figure 6C shows an example of another comprehensive study demonstrating the co-recruitment of six transcription factors in addition to p65 (RelA) to the enhancer and promoter regions of the LPS-inducible murine *Il1a* gene in dendritic cells, nicely demonstrating the convergence of different signaling pathways at the level of chromatin [50]. The interplay of the activities of all these transcription factors will ultimately shape the NF-κB-driven output of *Il1a* gene expression, which may also provide a plausible explanation why depletion of a NF-κB subunit must not necessarily result in a complete loss of target gene expression.

Noteworthy, both examples represent typical high-ON situations of the canonical NF-κB pathway as described in Figure 1A.

### 2.7. Detection of NF-κB-Mediated Gene Expression beyond mRNA

A direct read-out for active NF-κB is the induction of target gene expression, as shown by the RNA-seq analyses displayed in Figure 6B,C. Although NF-κB-mediated inhibition of gene expression has been observed in some cases, this transcription factor typically functions as an inducer of gene expression, as revealed by the comparative analysis of wild type cells and derivatives lacking NF-κB family members [62,65,84,107,108]. The immediate transcriptional activity can be determined by reporter gene experiments where a truncated promoter controlled by several κB binding sites drives the expression of a reporter gene such as luciferase or destabilized GFP. These experiments can give a quick answer concerning the relative NF-κB activation status, but the results may be biased by several technical aspects. Introduction of the plasmids into the target cell may constitute already a NF-κB-activating stimulus and the timing and the amplitude of NF-κB activity might be compromised. In addition, non-integrated transiently transfected reporter genes do not reflect the features of nuclear NF-κB regulation at the chromatin level. These potential pitfalls can nowadays be avoided by replacing endogenous NF-κB target gene regions by a reporter gene using CRISPR-Cas9 or further genome editing tools. Stable integration of reporter genes such as luciferase into the mouse genome has allowed the generation of NF-κB reporter mice that have yielded very important information on the NF-κB activity state in intact animals [109]. A straightforward strategy employs the direct determination of NF-κB target gene expression. Such assays can also be performed in a miniaturized format directly from limited or small numbers of cultured cells, which is possible even without RNA purification using appropriate kits [110]. We recommend the simultaneous quantification of several NF-κB target genes, which will provide a good estimate of the relative NF-κB activation status and will also provide information on the additional factors that shape the expression of individual NF-κB target genes. These target genes should ideally include the IκBα encoding *NFKBIA* gene, as in our hands the basal expression of this gene is induced by 10–30-fold by triggers of the canonical NF-κB pathway across different cell types, whereas the relative induction of other NF-κB target genes (such as chemokines) can be more variable, depending on the basal NF-κB activation status and activation of cooperating pathways. In general, the target genes to be analyzed must be carefully defined beforehand, as gene expression can be specific for the nature of the cell type, the physiological situation or the stimulus.

In this context, it should also be kept in mind that post-transcriptional regulation also contributes to NF-κB-inducible mRNA expression comprising further regulatory control at many levels including mRNA stability, localization, decapping, and deadenylation [111,112,113]. In addition, proinflammatory signaling cascades also regulate ribosomal translation and multiple steps of protein secretion [114,115,116,117,118]. Secreted NF-κB targets (IL-8, CXCL2, CCL20, IL-6) can also be quantitatively analyzed by antibody-based ELISA or multiplexed variants such as proximity extension assays (PEA) [119].

We used PEA technology to re-analyze the supernatants of cells with genome-edited mutations that either inactivated the major NF-κB-binding site in the human *IL8* enhancer (as marked in Figure 6B) or were introduced to generate human knockouts of the p65 protein ([62]). As shown in Figure 6D, the *CXCL8* enhancer most strongly regulates its next adjacent protein-coding gene, i.e., *CXCL8* (*IL8)*, but also affects the regulation of several other NF-κB target genes such as *CCL20*, *CXCL1, IL6,* or *CSF1*, most likely by an autocrine loop [62]. Notably, the p65 knockout does not result in complete loss of gene expression, which is often observed in such experiments and is explained by other pathways or the remaining NF-κB subunits also contributing to gene transcription of NF-κB target genes (Figure 6D). Altogether, these prototypic results demonstrate the complex and gene-specific phenotypes of NF-κB-dependent gene expression in unstimulated or stimulated situations. To judge the relevance of NF-κB activation in a given system, it is therefore advisable to not only demonstrate activation of the NF-κB pathway and the genomic recruitment of the subunits, but also to functionally validate the relevance of NF-κB for the final steps of gene expression. This includes analyzing the secretion of cytokines which may represent a further level of NF-κB regulation.

### 2.8. Advanced Analysis of NF-κB Activation States: Single Cell Detection of NF-κB Protein/Protein Interactions Together with mRNA Expression of NF-κB Target Genes

**Figure 7:**
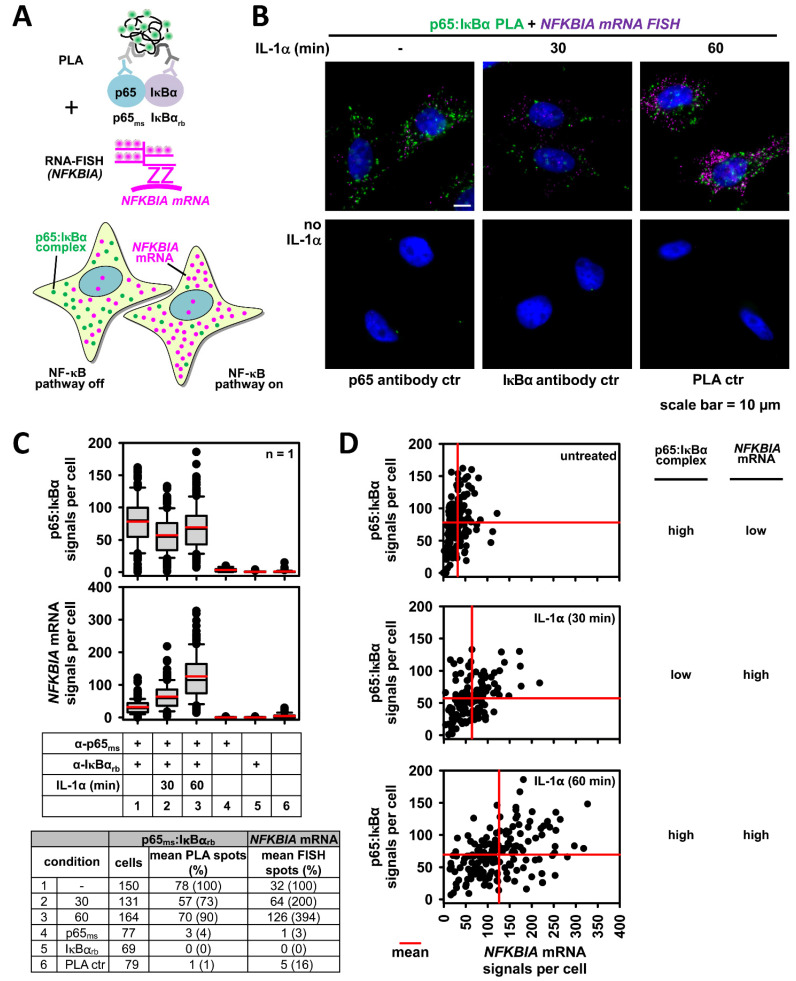
Advanced analysis of NF-κB activation: Monitoring key steps of cytosolic and nuclear NF-κB pathways in individual cells by combined PLA-RNA-FISH assays. (**A**) Scheme of the modified PLA procedure coupled to smRNA-FISH that allows to discriminate p65/IκBα complex formation in unresponsive cells compared to (neighboring) cells showing the IL-1α-induced dimished occurrence of p65/IκBα complexes along with increased expression of *NFKBIA* mRNAs encoding the IκBα protein. (**B**) HeLa cells remained untreated or were stimulated for 30 or 60 min with IL-1α (10 ng/mL) as shown. Cells were fixed and p65/IκBα complexes were revealed by PLA with specific antibodies followed by *NFKBIA* smRNA-FISH. PLA signals appear in green, *NFKBIA* FISH signals in pink and nuclei in blue (stained with Hoechst 33342). The cells were analyzed by fluorescence microscopy, representative merged pictures and PLA-negative controls (from untreated cells) are shown. (**C**) Both, PLA and smRNA-FISH signals were counted by the Duolink^®^ImageTool. The box plots show the distribution of PLA or smRNA-FISH signals. The table summarizes all relevant single cell data. (**D**) For each cell the number of p65/IκBα complexes was plotted against the *NFKBIA* smRNA-FISH signals. Data from untreated and IL-1-stimulated conditions are depicted as separate scatter plots, red lines indicate mean signals. The figure and legend were reproduced from Figure 6 of [121] under creative common licenses.

The amount of NF-κB bound to IκBα can be determined by co-immunoprecipitation experiments, but also at the single cell level by proximity ligation assays (PLAs), which are schematically explained in Figure 7A. This technique is based on the binding of two different antibodies to neighboring proteins, followed by hybridization of secondary antibodies which are covalently coupled to short ssDNA molecules. After hybridization of connector oligos to join the PLA probes and their ligation, a closed circle DNA template is formed and amplified by DNA polymerase. Complementary detection oligos coupled to fluorochromes hybridize to repeating sequences in the amplicons and the fluorescent dyes can be detected by fluorescence microscopy and thus indicate the occurrence and intracellular localization of protein/protein interactions [120]. We used this methodology to detect p65/IκBα complexes mainly in the cytosol of cells, as revealed by fluorescence microscopy and its quantitative and statistical analysis [121]. In addition, dynamic changes were observed, as administration of IL-1α resulted in a significant decrease of p65/IκBα complexes, followed by the re-formation of these complexes 90 min after the addition of the stimulus [121]. This experimental approach also allowed to visualize p50/p65 dimers in the nucleus and can be used to detect any other interesting protein/protein complex and also post-translational modifications, provided that suitable antibodies from two different species are available. As shown in Figure 7, PLA can also be combined with smRNA-FISH to detect the amount and intracellular localization of mRNAs [121]. This experiment once again corroborates the notion of the single cell heterogeneity of NF-κB pathway activation, which is expected to crucially determine the tissue reaction to NF-κB-activating stimuli.

## 3. Concluding Remarks

Here we have discussed a repertoire of widely used and emerging methods that serve to determine NF-κB activity in cells. Box 1 highlights the assays and parameters necessary to reveal and quantify true activation of NF-κB and its functional relevance for gene expression. While robust activation can be determined in a straightforward manner, low NF-κB activities as they occur late during the termination phase or in the “low-ON” state are more difficult to assess and require the determination of quantifiable parameters such as gene expression profiles. The CRISPR-Cas9 toolbox now enables the establishment of methods that allow monitoring functions of endogenous NF-κB in intact cells including sophisticated CRISPR-dCas9 activation systems [62,122]. Advanced systems, such as the combined PLA/RNA-FISH method and the progress in molecular imaging can be used to reveal the NF-κB function and activity in single cells and also in diseased tissues. Application of these approaches will greatly advance our knowledge on the stochastic and cell type specific nature of the NF-κB response [123].

Box 1 **Recommended parameters to monitor canonical NF-κB activation.**

**Key parameters to determine NF-κB activation**

(1) Time-dependent and stimulus-induced degradation and re-synthesis of IκBα
(2) Increase in nuclear p50 and p65
(3) Binding of p50 or p65 to the promoter of NFKBIA (the gene encoding IκBα)
(4) Induced mRNA expression of NFKBIA and other prototypical NF-κB target genes (CXCL8, IL6, TNFAIP3)
(5) Impaired inducible mRNA expression in cells with reduced p65 or p50 protein levels
(6) smRNA-FISH (with or without p50 or p65 IF or p65/IκBα PLA)
(7) ChIP-seq to determine direct regulation of NF-κB target genes

**Additional parameters needed to interpret context-dependent NF-κB activation pathways and activities**

(1) Assessment of the activation status of other signaling systems (JNK, p38, ERK, JAK/STATs, ER stress)
(2) Phosphorylation of IKKα/IKKβ
(3) Phosphorylation of p65 at Ser536 or Ser468
(4) Chromatin accessibility of NF-κB sites in enhancers or promoters of target genes
(5) Motif analyses of NF-κB elements and composite elements
(6) Determination of repressive or active histone marks flanking NF-κB elements
(7) Co-recruitment of typical NF-κB interacting transcription factors
(8) Co-recruitment of transcriptional coactivators/repressors
(9) Genome-wide profiling of mRNA expression in cells lacking NF-κB subunits
(10) Application of protein kinase inhibitors, e.g., TAK1 or IKK inhibitors
